# Effects of Image Degradation on Deep Neural Network Classification of Scaphoid Fracture Radiographs: Comparison Study of Different Noise Types

**DOI:** 10.2196/65596

**Published:** 2026-01-22

**Authors:** Chihung Lin, Alfred P Yoon, Chien-Wei Wang, Tung Chao, Kevin C Chung, Chang-Fu Kuo

**Affiliations:** 1 Center for Artificial Intelligence in Medicine, Chang Gung Memorial Hospital, Taoyuan, Taiwan; 2 Department of Artificial Intelligence, Chang Gung University, Taoyuan, Taiwan; 3 Division of Plastic and Reconstructive Surgery, Section of Hand and Microvascular Surgery, University of California Davis Medical Center, Sacramento, CA, United States; 4 Comprehensive Hand Center, Michigan Medicine, Ann Arbor, MI, United States; 5 Division of Rheumatology, Allergy and Immunology, Center for Artificial Intelligence in Medicine, Chang Gung Memorial Hospital, No.5, Fuxing Street, Guishan District, Taoyuan City 333, Taoyuan, 333, Taiwan, 886 3328-1200; 6 College of Medicine, Chang Gung University, Taoyuan, Taiwan; 7 Division of Rheumatology, Orthopaedics and Dermatology, School of Medicine, University of Nottingham, Nottingham, United Kingdom

**Keywords:** scaphoid fractures, contrast-limited adaptive histogram equalization, CLAHE, image quality, peak signal-to-noise ratio, PSNR, structural similarity index measure, SSIM, neural network.

## Abstract

**Background:**

Deep learning models have shown strong potential for automated fracture detection in medical images. However, their robustness under varying image quality remains uncertain, particularly for small and subtle fractures, such as scaphoid fractures. Understanding how different types of image perturbations affect model performance is crucial for ensuring reliable deployment in clinical practice.

**Objective:**

This study aimed to evaluate the robustness of a deep learning model trained to detect scaphoid fractures in radiographs when exposed to various image perturbations. We sought to identify which perturbations most strongly impact performance and to explore strategies to mitigate performance degradation.

**Methods:**

Radiographic datasets were systematically modified by applying Gaussian noise, blurring, JPEG compression, contrast-limited adaptive histogram equalization, resizing, and geometric offsets. Model accuracy was evaluated across different perturbation types and levels. Image quality was quantified using peak signal-to-noise ratio and structural similarity index measure to assess correlations between degradation and model performance.

**Results:**

Model accuracy declined with increasing perturbation severity, but the extent varied across perturbation types. Gaussian blur caused the most substantial performance drop, whereas contrast-limited adaptive histogram equalization increased the false-negative rate. The model demonstrated higher resilience to color perturbations than to grayscale degradations. A strong linear correlation was found between peak signal-to-noise ratio–structural similarity index measure and accuracy, suggesting that better image quality led to improved detection. Geometric offsets and pixel value rescaling had minimal influence, whereas resolution was the dominant factor affecting performance.

**Conclusions:**

The findings indicate that image quality, especially resolution and blurring, substantially influences the robustness of deep learning–based fracture detection models. Ensuring adequate image resolution and quality control can enhance diagnostic reliability. These results provide valuable insights for designing more accurate and resilient medical imaging models under real-world variability.

## Introduction

Significant advances in computer vision have led to remarkable improvements in deep neural network (DNN) performance on tasks such as medical image classification [1]. Despite these achievements, DNN-based systems exhibit limited robustness compared with human perception, especially in the context of image perturbations and corruption [2]. Adversarial attacks can cause substantial misclassifications, and even minor noise can adversely affect performance [3-5]. Nevertheless, state-of-the-art classifiers appear to exhibit some ability to overcome random noise [6].

In real-world medical imaging, DNN model robustness is critical because low-quality images and noise are not uncommon; these issues include radiographic underexposures and motion artifacts as well as downstream processing or transfer distortions that can alter image fidelity and affect artificial intelligence (AI) performance [7,8]. In addition, real-world clinical workflows may involve low-resolution screen recapturing or smartphone-captured radiographs in urgent or resource-limited settings, which introduce compounded degradations, such as resizing, compression, and display-related artifacts [9]. Our previous work on a DNN model that detected both visible and occult scaphoid fractures demonstrated that it was possible to reliably detect fractures of small bones and to assist in the radiographic detection of occult fractures that are not visible to human observers [10]. This experience indicated that image preprocessing, file formatting, and data storage could negatively impact model performance. We discovered that image processing techniques introduced noise into input files, potentially misleading the DNN model. Such noise, often imperceptible to the human eye, can significantly affect model performance and potentially lead to incorrect diagnoses [11]. Consequently, evaluation of performance is vital in convolutional neural network (CNN)–based models that process noisy images; this evaluation poses frequent challenges in clinical settings. Prior studies have revealed that neural networks are most accurate when the data to be classified exhibit quality similar to that of the model training data; it was recommended that noise be injected into the training data to increase model robustness [12]. However, the addition of many possible noises to a training dataset is both computationally expensive and impractical. To the best of our knowledge, no study has investigated the impacts of different types of noise on neural networks designed for fracture classification when radiographs serve as inputs.

This study investigated the robustness of our CNN-based scaphoid fracture classification model when various types of image noise were present. To comprehensively evaluate model performance, we simulated real-world conditions that yield low-quality clinical images. Using fine-tuning techniques to create noisy samples, we sought to enhance model performance under noisy or degraded conditions, thereby mitigating the adverse effects of image corruption [7]. Additionally, we examined the impacts of specific forms of image degradation on model performance; we explored particular model vulnerabilities that might warrant further refinement. Our goals were to ascertain model accuracy when processing low-quality clinical images and to identify the types of noise that most confounded the model, thereby providing valuable insights to aid the development of more resilient DNN-based medical image classifiers appropriate for real-world applications.

## Methods

### Ethical Considerations

The study protocol was approved by the institutional review board of Chang Gung Memorial Hospital (202202256B0).

### Scaphoid Fracture Classification Model

As previously described in our earlier work [10], the scaphoid fracture classification model was built using an EfficientNetB1 [13] backbone and 240×240-pixel red, green, and blue (RGB) images with a classification threshold of 0.5. In a study by Yoon et al [10], the model was originally trained and validated using 3991 scaphoid fracture radiographs and 5542 normal scaphoid radiographs.

**Figure 1.**
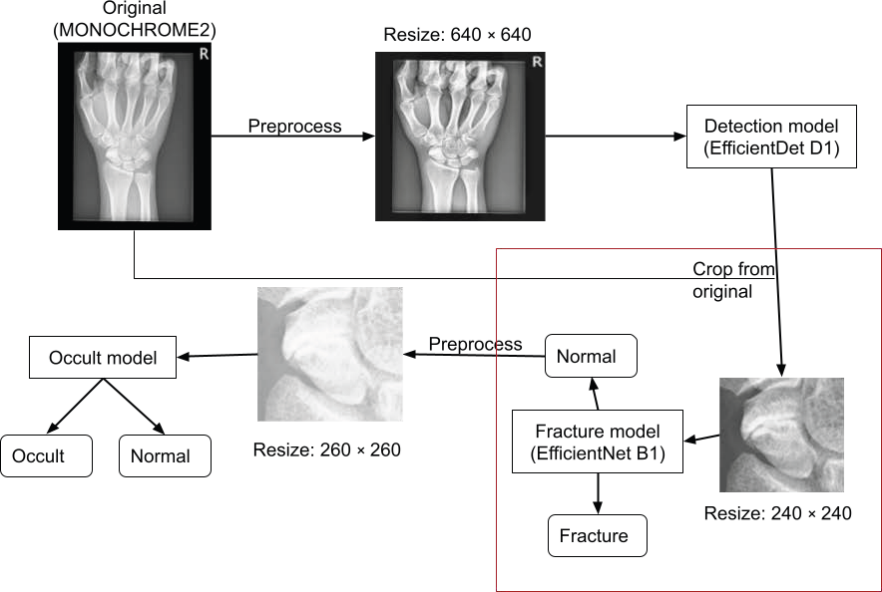
Complete pipeline for inference of a scaphoid fracture, including fracture (red box) and occult fracture. This report solely focuses on the performance of the fracture model.

In this study, we adopted the same model architecture as in the study by Yoon et al [10] and initialized the network with the finalized pretrained weights from that study, as shown in Figure 1. We then further fine-tuned this model using the 5286 training radiographs from the dataset described in the “Dataset and Preprocessing” section and evaluated it on an independent test set. Fine-tuning was performed using the AdamW optimizer, with appropriate adjustments to the learning rate, weight decay, and batch size. The learning rate was reduced if the validation loss failed to improve over 6 epochs, and training was stopped early when model performance did not increase further after 15 epochs.

### Dataset and Preprocessing

The scaphoid fracture classification model used in this study was fine-tuned and evaluated using 5954 radiographs stored in Chang Gung Memorial Hospital in Taiwan. Of these radiographs, 1483 were of fractured scaphoids, and the remaining 4471 were of normal scaphoids. Images were captured by commercially available x-ray machines from multiple manufacturers. All radiographs were reviewed by 3 experienced radiologists and labeled as *fractured* or *normal*. This dataset is distinct from the dataset used in our previous work [10], in which the model was originally developed using 3991 fracture and 5542 normal radiographs. In this study, we adopted the same model architecture as in the study by Yoon et al [10], initialized the network with the finalized pretrained weights from that study, and further fine-tuned the model using the 5286 training images described below before evaluating it on an independent test set.

The training dataset consisted of 5286 images, and the test dataset comprised 668 images. A total of 28 images were excluded for reasons including anatomical anomalies secondary to arthritis (12 images), 3 due to unclear laterality, 2 due to wrong-sided imaging, image artifacts introduced by the hardware (1 image), and imaging findings of likely previous fractures or chronic nonunion (10 images). Thus, the final test dataset contained 640 images. The images varied in size and position, but all were 12-bit grayscale images of posteroanterior views of the wrist. Most images were rectangular, with widths ranging from 1000 to 1600 pixels, and heights ranging from 1600 to 2200 pixels. If the photometric interpretation of an image was MONOCHROME1, the image was converted to MONOCHROME2. The scaphoid was isolated from each hand radiograph using a bounding box generated by a separate scaphoid detection model. This detector (not to be confused with the scaphoid fracture classifier) was not the focus of this study because its performance is robust against common perturbations.

### Rescaling Images to 8-Bit Depth

All radiographs were 12-bit grayscale posteroanterior wrist views that varied in terms of size and position, with widths between 1000 and 1600 pixels, and heights between 1600 and 2200 pixels. The images were rescaled to 8-bit grayscale and compiled into RGB images using the value of interest (VOI) lookup table and windowing operations that were also used during model training.

The DICOM images were stored in 12-bit grayscale, but the model accepted only 24-bit RGB (color) images. Thus, the images were first rescaled to 8-bit grayscale (with pixel values ranging from 0 to 255) and then compiled into RGB images for interpretation. This rescaling process was based on the maximum and minimum values of the image, rather than the actual bit depth. A VOI lookup table and windowing operations were applied to all images to adjust the pixel values based on the DICOM VOI LUT and windowing tags (pydicom. pixel_data_handlers.apply_voi_lut, version 1.4, 2022).

Differences emerge when images do not contain the lowest and highest pixel values. For 12-bit grayscale images, these pixel values are 0 and 4095, respectively. The resulting difference can brighten or darken the entire image. Furthermore, the use of a VOI lookup process may change the brightness and contrast. These differences are minor and usually imperceptible to the human eye.

### Generating Datasets for Noise Testing

#### Overview

To assess model performance in the presence of distortions, we generated several low-quality datasets using different noise perturbations. First, we read the test images using method 1. Images were cropped to the scaphoid regions demarcated by the bounding boxes, resized to 240×240 pixels, converted to 8-bit grayscale, and saved in PNG format. We refer to these baseline images (without noise) as “clean” hereafter.

To systematically evaluate the robustness of our scaphoid fracture classification model, we created multiple “noisy” or “degraded” datasets by applying a variety of image perturbations to our *clean baseline test set *of 640 scaphoid radiographs. Each perturbation type and severity level corresponds to a row in Table 1, which lists the name of the treatment, the number of images, and the specific operation performed. Next, we describe the main categories of perturbations and their implementations.

**Table 1.** Information on all datasets, including the datasets from which they were modified, sample counts, and methods of modification.

Treatment name	Modified from	N	Operation
Clean dataset	Original DICOM file	640	Read original DICOM file with (1)
Gaussian blur_0.5	Scaphoid	640	Apply Gaussian blur (σ=0.5) to the cropped scaphoid
Gaussian blur_1.0	Scaphoid	640	Apply Gaussian blur (σ=1.0) to the cropped scaphoid
Gaussian blur_1.5	Scaphoid	640	Apply Gaussian blur (σ=1.5) to the cropped scaphoid
Gaussian blur_2.0	Scaphoid	640	Apply Gaussian blur (σ=2.0) to the cropped scaphoid
Gaussian blur_2.5	Scaphoid	640	Apply Gaussian blur (σ=2.5) to the cropped scaphoid
Gaussian blur_3.0	Scaphoid	640	Apply Gaussian blur (σ=3.0) to the cropped scaphoid
Gaussian noise (RGB)_1	Scaphoid	640	Add RGB Gaussian noise with SD 1.00 to the cropped scaphoid
Gaussian noise (RGB)_2	Scaphoid	640	Add RGB Gaussian noise with SD 2.00 to the cropped scaphoid
Gaussian noise (RGB)_3	Scaphoid	640	Add RGB Gaussian noise with SD 3.00 to the cropped scaphoid
Gaussian noise (RGB)_4	Scaphoid	640	Add RGB Gaussian noise with SD 4.00 to the cropped scaphoid
Gaussian noise (RGB)_5	Scaphoid	640	Add RGB Gaussian noise with SD 5.00 to the cropped scaphoid
Gaussian noise (RGB)_6	Scaphoid	640	Add RGB Gaussian noise with SD 6.00 to the cropped scaphoid
Gaussian noise (grayscale)_1	Scaphoid	640	Convert image to grayscale, add Gaussian noise with SD 1.00 to the cropped scaphoid
Gaussian noise (grayscale)_2	Scaphoid	640	Convert image to grayscale, add Gaussian noise with SD 2.00 to the cropped scaphoid
Gaussian noise (grayscale)_3	Scaphoid	640	Convert image to grayscale, add Gaussian noise with SD 3.00 to the cropped scaphoid
Gaussian noise (grayscale)_4	Scaphoid	640	Convert image to grayscale, add Gaussian noise with SD 4.00 to the cropped scaphoid
Gaussian noise (grayscale)_5	Scaphoid	640	Convert image to grayscale, add Gaussian noise with SD 5.00 to the cropped scaphoid
Gaussian noise (grayscale)_6.0	Scaphoid	640	Convert image to grayscale, add Gaussian noise with SD 6.00 to the cropped scaphoid
JPEG compression_10	Scaphoid	640	Degrade scaphoid images with compression strength=10 (slightest)
JPEG compression_30	Scaphoid	640	Degrade scaphoid images with compression strength=30
JPEG compression_50	Scaphoid	640	Degrade scaphoid images with compression strength=50
JPEG compression_70	Scaphoid	640	Degrade scaphoid images with compression strength=70
JPEG compression_90	Scaphoid	640	Degrade scaphoid images with compression strength=90 (strongest)
CLAHE^a^_1	Scaphoid	640	Apply CLAHE (clip limit=1.0, tile grid size=[8, 8]) to the cropped scaphoid
CLAHE_2	Scaphoid	640	Apply CLAHE (clip limit=2.0, tile grid size=[8, 8]) to the cropped scaphoid
CLAHE_3	Scaphoid	640	Apply CLAHE (clip limit=3.0, tile grid size=[8, 8]) to the cropped scaphoid
CLAHE_4	Scaphoid	640	Apply CLAHE (clip limit=4.0, tile grid size=[8, 8]) to the cropped scaphoid
CLAHE_5	Scaphoid	640	Apply CLAHE (clip limit=5.0, tile grid size=[8, 8]) to the cropped scaphoid
Resize_400	Whole image	640	Resize whole images by width=400 while keeping the aspect ratio and crop out the scaphoid by projecting the recorded bounding box coordinates
Resize_600	Whole image	640	Resize whole images by width=600 while keeping the aspect ratio and crop out the scaphoid by projecting the recorded bounding box coordinates
Resize_800	Whole image	640	Resize whole images by width=800 while keeping the aspect ratio and crop out the scaphoid by projecting the recorded bounding box coordinates
Resize_1000	Whole image	640	Resize whole images by width=1000 while keeping the aspect ratio and crop out the scaphoid by projecting the recorded bounding box coordinates
Resize_1200	Whole image	640	Resize the whole images by width=1200 while keeping the aspect ratio and crop out the scaphoid by projecting the recorded bounding box coordinates
Resize_1400	Whole image	640	Resize whole images by width=1400 while keeping the aspect ratio and crop out the scaphoid by projecting the recorded bounding box coordinates
Geometrics_1	Whole image	5120	Modify the center point of bounding box (bbox) x,y coordinates by −20% and 0% and 20% of bbox length
Geometrics_2	Whole image	5120	Modify the center point of bbox x,y coordinates by −10% and 0% and 10% of bbox length
Geometrics_3	Whole image	10,240	Modify the 4 bbox x,y coordinates (xmin, xmax, ymin, ymax) by −10% and 10% of bbox length
Geometrics_4	Whole image	10,240	Modify the 4 bbox x,y coordinates (xmin, xmax, ymin, ymax) by −5% and 5% of bbox length
12-bit-rescale_1	Original DICOM file	640	Read original DICOM file using method (2) and crop the scaphoid with the recorded bbox coordinates
12-bit-rescale_2	Original DICOM file	640	Read original DICOM file using method (2) and crop the scaphoid with the detector
Screenshot_MicroDicom_1	Original DICOM file	640	Read DICOM files using MicroDicom with the default settings, screenshoot the whole image at a resolution of 550×780, and crop the scaphoid with the detector
Screenshot_MicroDicom_2	Original DICOM file	640	Read DICOM files using MicroDicom with the default settings, enter fullscreen mode, screenshoot the whole image at a resolution of 900×1050 and crop the scaphoid with the detector
Screenshot_ImageJ	Original DICOM file	640	Read DICOM files using ImageJ, adjust contrast to 12-bit, screenshoot the whole image at a resolution width of 600 while retaining the height-width ratio, and crop the scaphoid with the detector

a CLAHE: contrast-limited adaptive histogram equalization.

#### Clean Dataset

We refer to the original 640 cropped scaphoid images (taken directly from DICOM files and converted to 8-bit depth) as the “clean” dataset (row 1 in Table 1). This set serves as our baseline for comparison.

#### Gaussian Blur

We simulated *blur* by convolving each scaphoid region with a Gaussian kernel using SDs (σ) ranging from 0.5 to 3.0 (rows 2-7 in Table 1). Specifically, we used a Python image-augmentation library (eg, *imgaug*; Python Software Foundation) to apply GaussianBlur(σ=x). Each σ setting generated a separate dataset of 640 images.

#### Gaussian Noise

For grayscale noise (rows 8-13 in Table 1), we converted each cropped scaphoid image to 8-bit grayscale, then added random Gaussian noise with SD values in the set {1, 2, 3, 4, 5, 6}. The resulting noised images were reconverted to RGB (by replicating the grayscale channel 3 times) to match the model’s 3-channel input requirement.

For color (RGB) noise (rows 14-19 in Table 1), we similarly added Gaussian noise to the 3 RGB channels, resulting in “colored” noise. SD values were identical (1-6). Each level produced 640 modified images.

#### JPEG Compression

We degraded the quality of the cropped scaphoid images by JPEG compression (rows 20-24 in Table 1), with a compression strength of 10, 30, 50, 70, and 90 (higher values indicating more severe compression in our chosen library). Each compression level formed a dataset of 640 images.

This process simulates the impact of lossy image storage on fracture detection performance.

#### Contrast-Limited Adaptive Histogram Equalization

Contrast-limited adaptive histogram equalization (CLAHE; rows 25-29 in Table 1) enhances local contrast in radiographs, potentially exaggerating edges and intensifying noise. We generated 5 datasets by applying OpenCV’s createCLAHE() with *cliplimit* in {1.0, 2.0, 3.0, 4.0, 5.0} and tileGridSize=(8,8). Each CLAHE setting yielded 640 images.

#### Resizing Whole Images

Before cropping the scaphoid region, we resized the *entire* original wrist radiograph (rows 30-35 in Table 1) to widths of 400, 600, 800, 1000, 1200, or 1400 pixels (preserving the aspect ratio), then reapplied our scaphoid bounding box coordinates to crop out the scaphoid. Finally, the cropped regions were resized to 240×240 pixels for model input. This procedure mimics variations in image resolution and scaling during clinical acquisition or display.

#### Geometric Offsets

To examine the robustness of our model to bounding box inaccuracies, we systematically shifted or distorted the bounding box coordinates by ±5%, 10%, or ±20% of the bounding box size. This sometimes resulted in only partial scaphoid capture. Four separate datasets (Geometrics_1 to Geometrics_4) covered different offset ranges (rows 36-39 in Table 1). Each row includes multiple transformations, so the total number of images can exceed 640.

#### Twelve-bit Rescaling

Instead of converting the DICOM images with our typical method (method 1), we used an alternative approach (method 2) that directly scales 12-bit raw pixel values (0-4095) into 8-bit (0-255). We generated 2 variations: (1) 12-bit-rescale_1: recorded bounding box coordinates were applied to these rescaled images; (2) 12-bit-rescale_2: we reran the scaphoid detection model on these rescaled images to obtain new bounding boxes.

#### Screenshot Datasets

We opened the original DICOM files in different DICOM viewing software (eg, MicroDicom; ImageJ developed by National Institutes of Health and the Laboratory for Optical and Computational Instrumentation), adjusted the default display or resolution, and took screenshots of the entire wrist X-ray. Screen resolutions varied (eg, 550×780 or 900×1050; rows 42-44 in Table 1). We then cropped out the scaphoid region using our detection model, resizing the final images to 240×240. Each screenshot setting introduced different display parameters, simulating suboptimal clinical scenarios where images may be shared or interpreted via screenshots instead of original DICOM files.

Each of these modifications produced a new dataset of 640 images (except when multiple bounding box transformations were applied, resulting in a large number of images). Collectively, these datasets allowed us to evaluate the effects of image degradations on the classification model’s performance. Table 1 provides a concise summary of all transformations, whereas Multimedia Appendix 1 contains more detailed code snippets and pseudocode for each operation.

**Figure 2.**
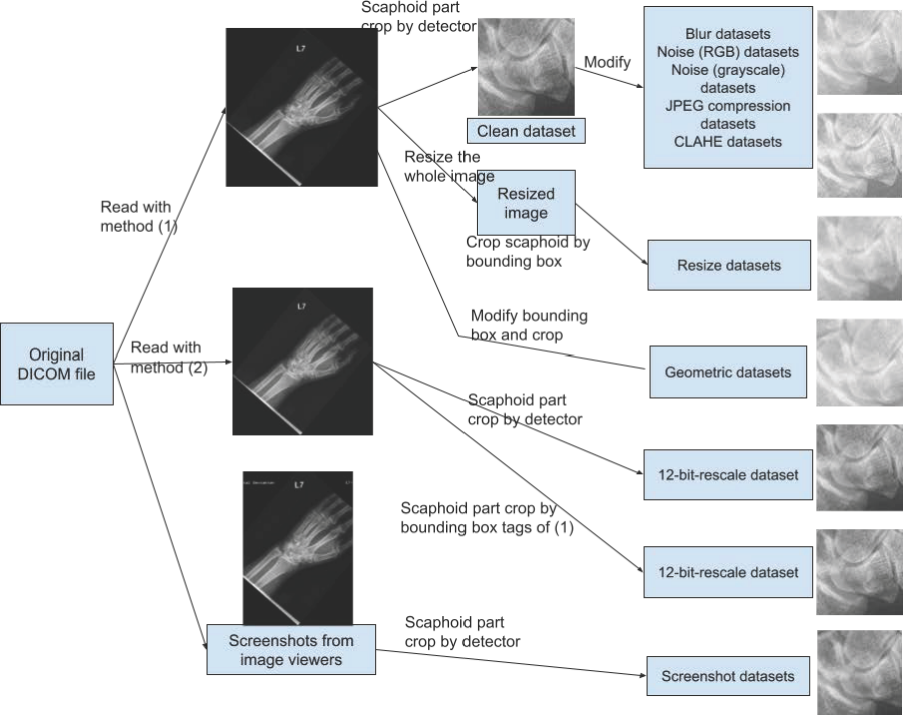
Flowchart of the pipeline used to generate clean and noisy datasets. CLAHE: contrast-limited adaptive histogram equalization;

Additionally, 5 datasets were produced using the rescale method or by capturing screenshots from the original DICOM files on image viewers. Figure 2 and Table 1 detail all 44 perturbations. The details of noise introduction into the dataset are described in Multimedia Appendix 1.

### Image Quality Assessment

The peak signal-to-noise ratio (PSNR) and the structural similarity indexing method (SSIM) [14,15] were used as image quality assessment methods when gauging alterations in images. PSNR is a widely used metric that assesses the fidelity of an image compared with its original or uncompressed version. PSNR quantifies the difference between 2 images by calculating the ratio of the maximum possible power of the signal (the image) to the power of the noise (the error that is introduced). A higher PSNR value indicates better image quality because less noise is introduced during changes. In contrast, SSIM is a more advanced metric for measuring image quality that considers structural information, luminance, and contrast when comparing 2 images. SSIM calculates local similarities between the 2 images and combines them into a single score, ranging from −1 to 1, where a higher value indicates greater similarity between the images. Both PSNR and SSIM values were calculated using TensorFlow image module (TensorFlow Module: tf.image), which allows efficient and accurate computations of both metrics. However, neither PSNR nor SSIM can be used for geometric transformations, such as affine and rotational adjustments, because both rely on pixel-by-pixel comparisons. Such comparisons become less meaningful when the spatial arrangement of image content is altered via geometric transformations. Consequently, datasets with modifications to the labeled scaphoid regions were excluded from comparison to maintain assessment integrity.

We calculated and compared PSNR and SSIM values for the clean and noisy images. Importantly, neither assessment is amenable to geometric transformations, such as affine and rotational adjustments. Therefore, the following datasets with modifications to the labeled scaphoid regions were excluded from the PSNR and SSIM comparisons: the 4 geometric datasets, the 12-bit rescale_2 datasets, and the 3 screenshot datasets. Both PSNR and SSIM were calculated using TensorFlow image functions (PSNR and SSIM).

### Model Evaluation and Performance Metrics

The scaphoid fracture classification model was evaluated by analyzing its performance on various test datasets that included clean images and distorted images with diverse noise levels. Performance metrics, including model accuracy, sensitivity, specificity, and *F*_1_-score, were calculated by comparing the model predictions with ground truth labels provided by experienced orthopedic and hand surgeons.

### Robustness Against Image Quality Degradation

To assess model robustness against image quality degradation, model performances on distorted images were compared with model performances on clean images. This comparison sought to estimate fracture classification accuracy when perturbations were present, offering insights into potential real-world applications of the model and its robustness when image quality varies in clinical settings.

### The Environment

Model inferences were executed on Linux Ubuntu 18.04 LTS (GPU: NVIDIA GeForce RTX 3080 (10 GB); Python version 3.7.13). Implementation was conducted with a TensorFlow backend and TensorFlow version 2.9.1.

## Results

### Performance of the Deep Learning Model With Varied Perturbations

We investigated the efficacy of a deep learning model designed to detect scaphoid fractures in radiographs, specifically when image quality had been deliberately compromised by adding perturbations. The primary datasets were altered using various methods: addition of Gaussian noise, blurring, JPEG compression, CLAHE, resizing, and geometric adjustments. Table 2 presents the quality assessment and model performance results across the evaluated datasets. Specifically, datasets that underwent blurring, Gaussian noise addition, JPEG corruption, CLAHE, or resizing are reported in rows 1–35; geometric datasets in rows 36–39; bit-rescaled datasets in rows 40–41; and screenshot datasets in rows 42–44. The model achieved an accuracy of 92.03% on the original unaltered dataset, which thus served as the performance benchmark for other evaluations.

**Table 2.** The quality assessment and model performance results across the evaluated datasets.

Treatment	PSNR^a^, mean (SD)	Average SSIM^b^, mean (SD)	Accuracy	Precision	Recall
Clean dataset	Inf	1.0000	0.9203	0.915	0.9119
Gaussian blur _0.5	52.5622 (2.6778)	0.9975 (0.0010)	0.9031	0.8896	0.9017
Gaussian blur _1.0	42.7419 (2.5447)	0.9758 (0.0096)	0.8422	0.7836	0.9085
Gaussian blur _1.5	39.9809 (2.5143)	0.9545 (0.0172)	0.8078	0.7299	0.9254
Gaussian blur _2.0	37.2251 (2.3945)	0.9183 (0.0272)	0.7625	0.6819	0.9085
Gaussian blur _2.5	35.4838 (2.3231)	0.8842 (0.0356)	0.7250	0.6530	0.8610
Gaussian blur _3.0	34.8328 (2.3077)	0.8674 (0.0399)	0.7203	0.6526	0.8407
Gaussian noise (grayscale)_1	47.7838 (0.1157)	0.9917 (0.0018)	0.9109	0.9190	0.8847
Gaussian noise (grayscale)_2	42.0190 (0.0432)	0.9697 (0.0066)	0.8906	0.8840	0.8780
Gaussian noise (grayscale)_3	38.5493 (0.0308)	0.9357 (0.0135)	0.8688	0.8436	0.8780
Gaussian noise (grayscale)_4	36.0689 (0.0271)	0.8929 (0.0216)	0.8422	0.7994	0.8780
Gaussian noise (grayscale)_5	34.1382 (0.0267)	0.8444 (0.0298)	0.8172	0.7602	0.8814
Gaussian noise (grayscale)_6	32.5600 (0.0276)	0.7932 (0.0376)	0.7734	0.7027	0.8814
Gaussian noise (RGB)_1	47.7851 (0.1134)	0.9917 (0.0018)	0.9156	0.9288	0.8847
Gaussian noise (RGB)_2	42.0211 (0.0368)	0.9697 (0.0065)	0.9094	0.9100	0.8915
Gaussian noise (RGB)_3	38.5487 (0.0225)	0.9357 (0.0135)	0.8969	0.8962	0.8780
Gaussian noise (RGB)_4	36.0686 (0.0180)	0.8929 (0.0215)	0.8891	0.8784	0.8814
Gaussian noise (RGB)_5	34.1376 (0.0173)	0.8444 (0.0299)	0.8547	0.8279	0.8644
Gaussian noise (RGB)_6	32.5599 (0.0168)	0.7931 (0.0376)	0.8219	0.7701	0.8746
JPEG compression_10 (slightest)	44.5076 (1.7566)	0.9829 (0.0046)	0.9141	0.9255	0.8847
JPEG compression_30	40.8137 (1.6190)	0.9619 (0.0091)	0.8922	0.9154	0.8441
JPEG compression_50	38.7102 (1.4519)	0.9411 (0.0119)	0.8688	0.8893	0.8169
JPEG compression_70	36.7313 (1.3293)	0.9125 (0.0150)	0.8609	0.8732	0.8169
JPEG compression_90 (strongest)	32.6132 (1.1419)	0.8146 (0.0233)	0.7656	0.6845	0.9119
CLAHE^c^_1	27.5678 (1.9698)	0.932 (0.0095)	0.8875	0.9176	0.8305
CLAHE_2	21.3350 (1.4853)	0.7836 (0.0182)	0.8250	0.9256	0.6746
CLAHE_3	18.7906 (1.3643)	0.6860 (0.0220)	0.7953	0.9457	0.5898
CLAHE_4	16.6896 (1.2363)	0.5904 (0.0262)	0.7812	0.9641	0.5458
CLAHE_5	15.6361 (1.1716)	0.5395 (0.0306)	0.7703	0.9353	0.5390
Resize_400	27.4436 (2.8561)	0.6578 (0.1013)	0.7188	0.6353	0.9153
Resize_600	29.2871 (3.0279)	0.7108 (0.1042)	0.8172	0.7514	0.9017
Resize_800	30.8330 (3.07574)	0.7651 (0.1007)	0.8516	0.8106	0.8847
Resize_1000	32.3258 (3.6219)	0.8130 (0.0932)	0.8672	0.8454	0.8712
Resize_1200	33.3882 (3.8631)	0.8432 (0.08724)	0.8797	0.8682	0.8712
Resize_1400	34.5316 (3.6730)	0.8754 (0.0748)	0.8938	0.8874	0.8814
Geometrics_1	N/A^d^	N/A	0.8635	0.8524	0.8513
Geometrics_2	N/A	N/A	0.8949	0.9003	0.8682
Geometrics_3	N/A	N/A	0.8827	0.8765	0.8678
Geometrics_4	N/A	N/A	0.9021	0.9041	0.8809
12-bit-rescale_1	48.0188 (8.0564)	0.9945 (0.0181)	0.9188	0.9119	0.9119
12-bit-rescale_2	N/A	N/A	0.9141	0.9027	0.9119
Screenshot_MicroDicom_1	N/A	N/A	0.8281	0.7666	0.9017
Screenshot_MicroDicom_2	N/A	N/A	0.8625	0.8439	0.8610
Screenshot_ImageJ	N/A	N/A	0.7812	0.7123	0.8814

a PSNR: peak signal-to-noise ratio.

b SSIM: structural similarity index measure.

c CLAHE: contrast-limited adaptive histogram equalization.

d N/A: not applicable.

### The Effects of Noise and Perturbations

This section discusses model performance on datasets directly modified from the cropped scaphoid radiographs and resized datasets without geometric modifications. Table 2 presents the quality assessment and model performance results across the evaluated datasets. Specifically, datasets that underwent blurring, Gaussian noise addition, JPEG corruption, CLAHE, or resizing are reported in rows 1–35; geometric datasets in rows 36-39; bit-rescaled datasets in rows 40-41; and screenshot datasets in rows 42-44. Further details can be found in Multimedia Appendix 1.

For datasets that included cropped scaphoid radiographs and resized images, if the severity of image degradation was minimal, the effect on model accuracy was negligible. An important observation was that the model exhibited varying degrees of resilience against different image perturbations. Even in some datasets with similar PSNR and SSIM values and comparable image quality, some discrepancies in model performances were observed. These findings underscore the nuanced robustness of the deep learning model against different image distortions.

On some noisy datasets, such as Gaussian blur_0.5, Gaussian noise (RGB)_1, Gaussian noise (grayscale)_1, and JPEG compression_10, the model performances were similar to that on the clean dataset. These datasets had the least severe distortions; the pixel values changed minimally, as indicated by the high PSNR and SSIM values. However, as the perturbations increased in severity, image features deteriorated further, and model performance declined. This trend was observed across all treatments, although the extent of performance decline varied according to the type of perturbation. We conclude that if the severity of a perturbation can be maintained below a specific level in degraded images, the model can maintain good performance.

### Robustness Against Different Types of Perturbations

Next, we compared model robustness across different image perturbations. Although some datasets yielded similar average PSNR and SSIM assessments, model performances differed. This finding suggested that the model is more robust against certain types of distortions but more vulnerable to others.

#### Color and Grayscale Gaussian Noise

We expected that the PSNR and SSIM assessments would be similar after the introduction of Gaussian noise, regardless of whether the noise was in color or grayscale. This expectation was confirmed by the similar average PSNR and SSIM values of the corresponding RGB and grayscale Gaussian noise datasets. Nevertheless, model accuracy was considerably lower when grayscale noise was present, suggesting that the model solely enhanced resilience to color perturbations. This may be explained by the fact that the training set was exclusively composed of grayscale samples, although the model input layer accepted 3-channel color images.

#### Gaussian Blurring

Gaussian blurring substantially degraded model performance, comparatively more than other noises with similar image quality metrics. For images with PSNR values between 35 and 50, linear regression analysis revealed that Gaussian blurring was associated with a 5% to 12% decrease in accuracy compared with other nongeometric transformation techniques. Although blurring an image does not significantly decrease image quality, blurring likely affects fracture features and thus negatively affects detection. This finding is consistent with the results of a previous study, which concluded that neural networks are very sensitive to blurring, probably because textures and edges are removed [16-18].

#### JPEG Compression

The effect of JPEG compression on model performance was similar to the effect of grayscale Gaussian noise. The neural network was surprisingly resilient to JPEG compression. Even after a 90% file size reduction via JPEG compression, the model achieved an accuracy of greater than 81%. This finding has important clinical implications: DICOM images, which are often larger than 20 MB, can be compressed by up to 70% via JPEG, but the model will maintain 90% accuracy in terms of detecting scaphoid fractures. This capability will render model implementation more computationally efficient.

#### Resizing

The resizing perturbations were designed to simulate a realistic low-resolution workflow in which radiographs may be downsampled before region extraction and later rescaled for AI inference; therefore, this setting reflects the combined effects of downscaling information loss and upscaling or interpolation artifacts from small cropped regions of interest (ROIs), rather than a purely isolated resolution test. The downscaling treatments required the new image to store information using fewer pixels than the original, which forced the image to compress its content. As a result, a substantial amount of information was lost when the number of available storage units was reduced, leading to blurry images and lowered image quality, as demonstrated by the PSNR and SSIM values. However, fracture detection accuracy did not significantly decline until the PSNR values fell below 30. Compared with other noise treatments, resizing an image may adequately preserve the features required for fracture detection.

#### Contrast-Limited Adaptive Histogram Equalization

CLAHE treatment enhances image contrast and thus dramatically alters the image [19,20]. A CLAHE-enhanced image can inadvertently mislead the model, especially if the model has not been trained with CLAHE-augmented data. Model accuracy decreased as the parameter “clip limit” (maximum limit of the adaptive histogram equalization) increased, considerably lowering the PSNR and SSIM values. However, the poor metric values do not necessarily imply that CLAHE transformation worsens the model performance more severely than that of other perturbations. Even at a cliplimit of 2, the PSNR declined to 21, lower than the PSNR after any Gaussian blur or JPEG treatment, and the model accuracy decreased to 83%. As the cliplimit was subsequently increased, the accuracy precipitously fell to 77% (Table 2). This finding differs from the result of a previous study, in which neural networks were resilient against changes in image contrast [17]. This discrepancy may be because Dodge and Karam [5] investigated contrast reduction only via grayscale image superimposition and assessed correct image classification (eg, a dog and a cat) using network models. In contrast, a scaphoid DNN must detect subtle linear features when identifying fractures, and CLAHE likely obscures contrast along the fracture line. CLAHE is useful when enhancing x-ray images before human interpretation, but it should only be used in neural networks that are trained via CLAHE augmentation. Additionally, if a chosen medical imaging software exhibits built-in CLAHE-enhancing features, the neural network must be trained with CLAHE-augmented data.

### Differences in the Reductions of Precision and Recall Rates

The prevalence of scaphoid fractures in the test dataset was 46%. As all noisy datasets were derived from this dataset, the prevalences were identical. Accordingly, a decline in model accuracy can be attributed to either reduced precision or recall rates, with the former creating more false positives and the latter creating more false negatives.

In the last step of training, the model was fine-tuned to achieve precision and recall rates of 91%. We expected that these performance metrics would decrease when interpreting noisy datasets. Intriguingly, the severities of performance decline differed for precision and recall. Although most noisy datasets triggered declines in both precision and recall, the performance deteriorations exhibited by the precision rates were more pronounced. However, the recall rate was usually acceptable, even in heavily altered datasets. As the model seeks to identify all possibly useful scaphoid fractures, it remains clinically robust in terms of detecting fractures despite image perturbation, but at the cost of increased false-positive rates.

Only a few datasets showed the opposite, with a precision rate much greater than the recall rate; these were the JPEG compression_50, JPEG compression_70, and all CLAHE datasets. Conversely, the remaining JPEG compression datasets demonstrated a higher recall than precision rate, similar to other changes. Therefore, JPEG compression is likely not the principal explanation for such findings.

However, we found that CLAHE image treatment must be performed with caution. CLAHE primarily reduces recall (increasing false negatives) while precision tends to increase, indicating a heightened risk of missed fractures at higher clip limits (Table 2). If the images to be interpreted are of low quality and CLAHE enhancements are applied, machine learning scientists should be wary of inadvertently increasing the false-negative rates.

### Relationship Between Image Quality Assessments and Model Performance

We combined all noisy datasets (excluding the CLAHE datasets, given their heterogeneity) into a single dataset with 18,560 samples exhibiting various perturbations. We used this combined dataset to investigate the relationship between image quality and model performance.

We calculated 25 quantiles of SSIM value distributions and grouped the images according to quantile; this approach yielded 25 groups with 742 or 743 samples each. Our grouping method effectively randomized the images and eliminated the effect of any particular degradation treatment when grouping images from different datasets by image quality. We regrouped images by SSIM quantiles, rather than PSNR quantiles, because the distributions of SSIM values within each noisy dataset were wider and enabled easier stratification.

**Figure 3.**
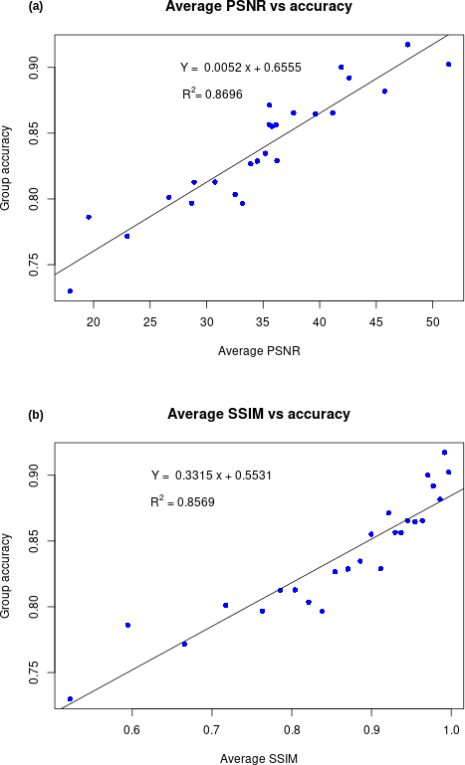
The images were divided into 25 groups according to the structural similarity index measure (SSIM) value quantiles. PSNR: peak signal-to-noise ratio.

For each group, the average PSNR, average SSIM, and model accuracy were calculated (Figure 3). We found a strong linear relationship between average image quality and model accuracy. The model performed better on images with higher quality assessments.

The average PSNR, average SSIM, and group accuracy for each were plotted; the accuracy exhibited strong linear relationships with both image quality assessments.

Both PSNR and SSIM regressions yielded high adjusted R-squared values (*P*<.001). As the PSNR and SSIM values are not independent, it is reasonable to expect that the 2 assessments would yield similar results for most image degradations [21]. However, the SSIM was normalized, whereas the PSNR was not. Robust linear associations between image quality evaluations and model performances were evident. Specifically, PSNR and SSIM, indicators of image quality, served as reliable independent tests that predicted AI performance under various perturbations. Higher image quality evaluations were invariably correlated with superior model performances. Although certain perturbations, such as geometric offsets or pixel value rescaling, did not appear to influence model performance, resolution was identified as a key factor, particularly in screenshot images. An enhanced focus on the ROI, such as zooming, effectively averted accuracy reduction. Recent research introduced a framework for the creation of Robust Medical Imaging AI models, which focuses on developing robust AI models for chest radiographs by addressing real-world sources of image degradation such as device heterogeneity, screen-captured inputs, and compression artifacts [22].

### Effects of Geometric Offsets

CNN models extract hierarchical features from local regions of an image through convolution and pooling operations. In principle, a moderate change in object location within the image should therefore not drastically confuse the model. Our results generally reflected this expectation: the 4 geometrically modified datasets retained good overall accuracy when the scaphoid remained fully within the field of view. However, accuracy declined as the strength of the affine transformations increased. A likely explanation is that parts of the scaphoid were shifted outside the image boundaries, resulting in cropped or incomplete scaphoid regions being presented to the model. Such extreme geometric distortions are unlikely to occur under real-world clinical conditions.

To further assess whether the detection model contributed to performance degradation under realistic settings, we evaluated its robustness on the 3 real-world screenshot datasets and the 12-bit-rescale_2 dataset and compared these results with those from the 4 geometric offset datasets. As summarized in Table 3, the mean intersection over union (IoU) values for the screenshot and 12-bit-rescale_2 datasets (0.8728, SD 0.0679 to 0.9836, SD 0.0268) were consistently higher than those of the geometric offset datasets (0.5752, SD 0.0952 to 0.8530, SD 0.0222). The geometric offset datasets (Geometrics_1‐4) were intentionally designed as synthetic baselines to simulate pure localization errors of the detector. The lower IoU values in Geometrics_1‐3 (0.5752‐0.7546) correspond to increasingly large artificial offsets, whereas Geometrics_4 (mean 0.8530, SD 0.0222) represents the least-modified geometric condition in which the scaphoid remains fully visible after offset treatment. Notably, Table 3 presents that the detector achieved IoU values exceeding Geometrics_4 on all screenshot datasets, suggesting that the detection model remains stable under realistic screenshot-related degradation.

**Table 3.** IoU values of the datasets which the detection model was involved with and the geometric datasets.

Datasets involve detection model	IoU^a^, mean (SD)
Screenshot_MicroDicom_1	0.8768 (0.0625)
Screenshot_MicroDicom_2	0.8728 (0.0679)
Screenshot_ImageJ	0.8907 (0.0554)
12-bit-rescale_2	0.9836 (0.0268)
Geometric datasets^b^	
Geometrics_1	0.5752 (0.0952)
Geometrics_2	0.7546 (0.0662)
Geometrics_3	0.7063 (0.0529)
Geometrics_4	0.8530 (0.0222)

a IoU: intersection over union.

b Considering geometric datasets IoU as baseline, the detection model performed better on all the screenshot datasets, showing its robustness toward perturbations, and that the performance of the detection model would not be a major factor leading to failure of the classification model when image degradation occurs.

As the classification model still maintained reasonable accuracy across the geometric baselines, IoU values comparable to or higher than Geometrics_4 indicate that under real-world screenshot- and rescaling-related perturbations, the detector’s localization accuracy is at least similar to its best geometric baseline performance and therefore is less likely to be a dominant contributor to the observed classification performance changes. Overall, these findings suggest that detector-related bounding box offsets likely play a limited role in the performance degradation observed in these realistic conditions.

### Effects of Pixel Value Rescaling Methods

The 2 methods for rescaling images from 12-bit to 8-bit did not materially affect model performance. The 2 datasets derived from the alternative rescaling method, 12-bit-rescale_1 and 12-bit-rescale_2, showed only a minor decrease in accuracy (<1%), although the rescaled images were nearly identical. Examination of the few inconsistent cases revealed that the classification confidence scores for “fracture” and “nonfracture” were much closer in these instances, with differences typically on the order of 10^2^. In contrast, in most other cases, the confidence score gap exceeded 10^3^, indicating stronger and more stable model decisions. These relatively uncertain confidence scores suggest that the inconsistent cases were inherently difficult for the model to judge, even without perturbation. As the model’s decisions for such cases were already unstable, slight modifications introduced by the alternative rescaling algorithms—despite causing only minimal pixel value changes—could flip the predicted label. This likely explains the minor performance differences observed among the clean dataset and the 2 rescaled datasets (12-bit-rescale_1 and 12-bit-rescale_2).

### Screenshot Datasets

Taking screenshots was the most complex perturbation of all the studied treatments. Resolution may be the factor that most strongly affects model performance. The only difference between the datasets Screenshot_MicroDicom_1 and Screenshot_MicroDicom_2 was the resolution; changing the resolution from 900×1050 to 550×780 pixels resulted in an accuracy reduction from 86.3% to 82.8%. The dataset Screenshot_ImageJ, the dataset with images resized to a width of 600 pixels while keeping the aspect ratio collected using ImageJ, exhibited the worst resolution and worst read accuracy (78.1%). Such declines in accuracy, precision rate, and recall rate were similar to declines observed in the corresponding resized datasets (Screenshot_MicroDicom_1 with Resize_600, Screenshot_MicroDicom_2 with Resize_1000, and Screenshot_ImageJ with Resize_600). Although the detection model had been involved in the preparation of these datasets, Table 3 implies that in these cases, scaphoids were correctly detected and cropped. In conjunction with the fact that the classification performance of screenshot datasets was worse than that of any of the geometric datasets, the degradation in model performance indeed stems from resizing effects. We also observed that although resolution deterioration was the major factor, the screenshot process did not impact model performance identically to pure resizing. Specifically, Screenshot_ImageJ showed a modest but consistent performance drop compared with its corresponding resized dataset (Resize_600) and also performed worse than another screenshot dataset (Screenshot_MicroDicom_1) collected at a similar resolution. This suggests that viewer-specific display or resampling algorithms during screenshot capture may also influence model performance.

On the basis of these results, one strategy that may prevent accuracy reduction after taking screenshots would be to zoom into the image and then take a screenshot at the 100% level. Although the radiographic image subsequently may not fit within the screen display, it is unnecessary to capture the entire image because the only ROI is the scaphoid.

### Computational Cost and Inference Time

We evaluated the average inference time by running through the 640 whole-hand x-ray images from the clean testing dataset using the complete preprocessing, bounding box detection, and classification inference pipeline. We obtained a computation time of approximately 6 to 8 ms per image on an NVIDIA RTX 3080 (10 GB RAM), while warmup runs were not included. The end-to-end pipeline used less than 200 MB GPU memory per 240×240×3 input (including framework overhead), enabling near real-time inference on a single RTX 3080. Consequently, our approach is compatible with near-real-time applications, assuming a well-optimized implementation. Although resizing or blurring images may incur a minor preprocessing overhead, these operations add only approximately 1 ms to the total inference time, indicating that real-time usage in clinical workflow is feasible on a modern GPU.

## Discussion

### Principal Findings

Taken together, our findings highlight that the deep learning model’s performance is inversely related to the severity of image degradation, with Gaussian blur, grayscale noise, and CLAHE standing out as the most disruptive factors. These types of distortions can destroy or obscure the crucial edge information that the network relies upon for detecting subtle fracture lines. In contrast, moderate JPEG compression, resizing, or color noise had a less pronounced effect. Notably, the resizing experiments in this study reflect a realistic low-resolution workflow involving downscaling, then cropping, and then upscaling, which is consistent with real-world recaptured or low-resolution imaging scenarios described in the literature [9]. While accuracy decreases were comparable between Screenshot_ImageJ and the corresponding Resize datasets, Screenshot_ImageJ showed a modest additional reduction in recall, suggesting that screenshot recapture can affect sensitivity beyond pure resizing.

From a clinical perspective, these results underscore the importance of maintaining sufficient image fidelity and avoiding overly aggressive postprocessing steps. If CLAHE-based enhancement or heavy contrast adjustments are used, training the model with matching augmentations may be necessary to preserve performance. Similarly, our analysis indicates that modest compression does not necessarily compromise diagnostic accuracy, suggesting an avenue for reducing file sizes without compromising model output.

Although the network proved fairly robust across various perturbations, investigators should remain cautious when applying transformations, such as extreme blurring, under or overexposure, or very low-resolution screenshots. In these scenarios, crucial details may be irretrievably lost, leading to significantly higher misclassification rates. Our results also emphasize that the precision rate is especially sensitive to noise, often dropping faster than recall. This behavior means that some perturbations can inflate false positives, which may still be acceptable in screening contexts that favor high recall but could impact workflows that depend on precise diagnoses.

In certain clinical scenarios, obtaining the original radiographic file may not be immediately feasible—such as in emergency settings, remote consultations, or when imaging systems have limited data access privileges. In such situations—particularly in emergency department workflows where the original radiographic DICOM file may not be readily accessible because of time pressure, remote consultation needs, or limited system privileges—clinicians or technicians may rely on on-screen screenshots or smartphone photographs for rapid review, sharing, or AI-assisted interpretation. Although practical, these recaptured images can introduce unintended degradation due to variations in capture resolution, scaling distortions, and secondary compression, which may obscure subtle fracture cues. This real-world practice motivated our inclusion of screenshot-based perturbations as a clinically relevant proxy for urgent or resource-limited conditions. To replicate such real-world conditions, our study incorporated a “screenshot” perturbation by capturing radiographs at different image sizes and resolutions. The resulting artifacts mimic the geometric and pixel-level distortions that may arise in urgent or resource-limited workflows, providing insight into how these factors influence model performance.

Although this study was conducted using data from a single institution, the dataset represents one of the largest and most diverse radiographic collections in Taiwan, drawn from multiple campuses within the Chang Gung Memorial Hospital system, with different scanners and protocols, introducing natural variability while maintaining consistent image quality and annotation standards. The main objective of this study was to test a methodological approach for evaluating model robustness to image degradation, rather than to claim broad generalization to all clinical environments. The single-institution design, therefore, provided a stable yet sufficiently varied dataset for controlled experimentation.

### Comparison With Prior Work

Our findings are consistent with prior studies showing that CNNs are sensitive to image degradation and domain shifts in medical imaging. Recent research introduced the RoMIA framework, which aims to develop robust AI models for chest radiographs by addressing real-world sources of image degradation, such as device heterogeneity, screen-captured inputs, and compression artifacts. In parallel, previous studies have shown that AI can enhance image quality and improve diagnostic reliability across multiple imaging modalities, including computed tomography and radiography [1,7,23]. Moreover, real-world deployments have demonstrated that recaptured or smartphone-captured radiographs introduce compounded degradations (eg, scaling, compression, and display artifacts) that can alter AI outputs, underscoring the clinical relevance of evaluating robustness under such workflows [9]. Taken together, these studies highlight the growing emphasis on image fidelity and consistent acquisition parameters as key factors for ensuring robust and generalizable AI performance. Previous research [18] has demonstrated that blurring and contrast alteration primarily affect the high-frequency components essential for delineating structural boundaries—similar to the degradation patterns observed in this study. The alignment between our results and those of earlier works reinforces that these degradation effects are likely intrinsic to CNN-based architectures rather than model-specific artifacts.

### Limitations

This study has several limitations. First, the robustness results reported here are relative to our standard clinical preprocessing pipeline. Specifically, the “clean” baseline was generated by detector-based cropping followed by resizing each scaphoid ROI to 240×240 pixels to match the fixed input size of EfficientNetB1. As this crop-and-resize step is itself a lossy operation, our robustness conclusions should be interpreted within this trained-and-deployed pipeline context, rather than as absolute performance relative to the raw, unprocessed DICOM images. Second, our analysis was confined to a single deep learning architecture, EfficientNetB1. Different backbones (eg, ResNet and DenseNet) may exhibit varying levels of robustness to specific noise types, such as Gaussian blur or CLAHE. Nevertheless, we expect that many of the general trends—such as vulnerability to edge-destroying blur—would hold true across CNN-based architectures. Third, all experiments were performed on retrospective datasets from a single institution and focused on controlled pixel-level perturbations. As a result, the data may not fully capture the diversity of real-world clinical acquisition conditions or additional artifacts, such as motion blur, partial occlusions, and multiview variability. Future prospective, multi-institutional studies incorporating these realistic factors would provide a more comprehensive assessment of model resilience and generalizability.

### Future Directions

Future work could extend our methodology by evaluating multiple architectures under identical perturbations to quantify differences in susceptibility and to determine whether certain network designs are inherently more robust. Incorporating multiview data and realistic clinical artifacts (eg, motion blur and underexposure) may also help develop more generalizable models. Additionally, advanced data augmentation strategies that simulate image degradation during training could enhance robustness. Exploring hybrid or ensemble models that integrate texture- and shape-based cues, as well as integrating domain-specific priors, could further mitigate the impact of low-quality inputs.

### Conclusions

Neural network models designed to complement radiographic interpretation in clinical practice will inevitably encounter image quality distortions due to variations in acquisition, processing, and storage. In this study, we systematically evaluated the effects of image degradation on the performance of a DNN for scaphoid fracture classification. We found a strong negative correlation between image quality and model accuracy, with Gaussian blur, grayscale Gaussian noise, and CLAHE exerting the greatest influence on performance.

Performance decline was primarily driven by decreases in precision, whereas recall remained relatively stable. When developing neural networks for fracture detection in clinical radiography, training with targeted perturbations—particularly Gaussian blur, grayscale noise, and CLAHE—may improve the model robustness and ensure more reliable performance in diverse clinical environments.

## Supplementary material

10.2196/65596 Multimedia Appendix 1 Representative examples of all image perturbation types evaluated in this study.

## Abbreviations

AI: artificial intelligence
CLAHE: contrast-limited adaptive histogram equalization
CNN: convolutional neural network
DNN: deep neural network
IoU: intersection over union
PSNR: peak signal-to-noise ratio
RGB: red, green, and blue
ROI: region of interest
SSIM: structural similarity index measure
VOI: value of interest
