# Supplementary material for: Effects of Image Degradation on Deep Neural Network Classification of Scaphoid Fracture Radiographs: Comparison Study of Different Noise Types
Source: JMIR Med Inform. 2026 Jan 22;14:e65596. doi: 10.2196/65596 (PMC12826633; doi:10.2196/65596)
Supplement: Multimedia Appendix 1 [file medinform-v14-e65596-s001.docx]

**Multimedia Appendix 2**

**Gaussian Blur**

Blur the images by Gaussian kernel [16, 18] with sigma ∈ {0.5, 1.0, 1.5, 2.0, 2.5, 3.0}. Each severity forms a noisy dataset.

We used a python package, *imgaug*, for this process.(Function located at https://imgaug.readthedocs.io/en/latest/source/overview/blur.html#gaussianblur)


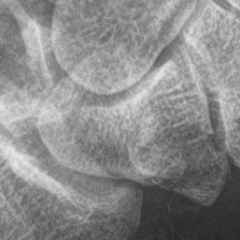

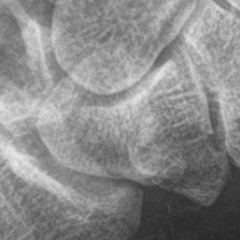

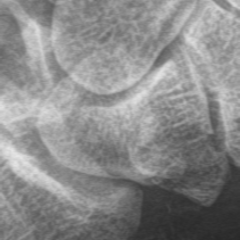

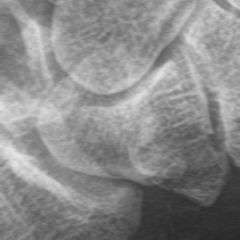

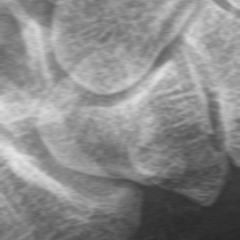

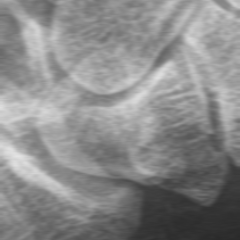

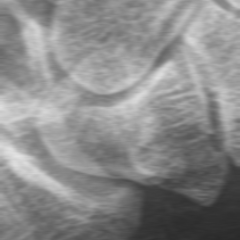


Clean dataset

Blur

sigma = 0.5

sigma = 0.5

sigma = 0.5

sigma = 0.5

sigma = 0.5

sigma = 0.5

**Gaussian Noise (RGB)**

Add Gaussian noise to the scaphoid images.

This distortion was carried out with ImageJ. We read the cropped scaphoids, turned them into RGB Colorspace, and applied Gaussian noise by running ”***Add Specified Noise...***” with standard deviation ∈ {1.00, 2.00, 3.00, 4.00, 5.00, 6.00}.

Since the colorspace was RGB, the Gaussian noise was also colored.


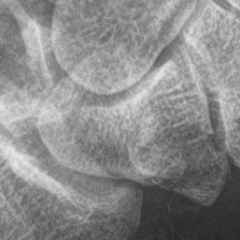


Clean dataset

noise (RGB)

std = 1.00

std = 4.00

std = 5.00

std = 2.00

std = 3.00

std = 6.00


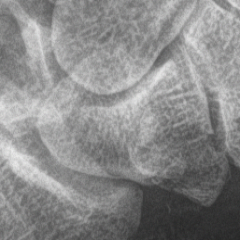

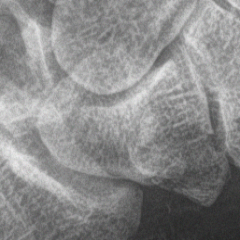

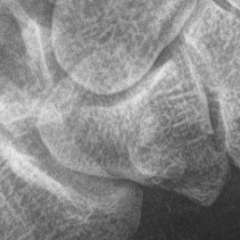

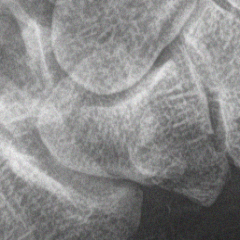

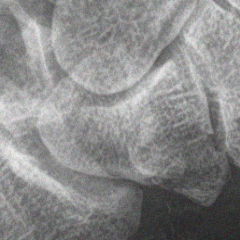

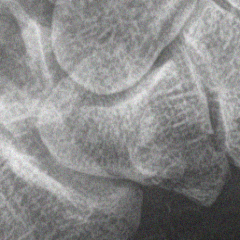


**Gaussian Noise (grayscale)**

The process to generate grayscale Gaussian noise datasets was similar to those of RGB Gaussian noise, except that the images were converted not to RGB colorspace but to 8-bit grayscale. Thus, the noise added was in grayscale as well. The noisy grayscale images were converted back to RGB and saved.

We set standard deviation ∈ {1.00, 2.00, 3.00, 4.00, 5.00, 6.00}.


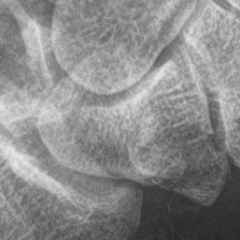


Clean dataset

noise (grayscale)

std = 1.00

std = 4.00

std = 5.00

std = 2.00

std = 3.00

std = 6.00


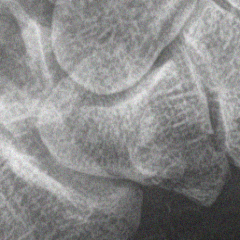

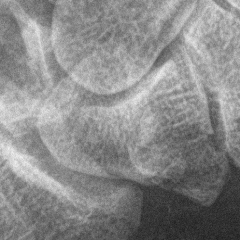

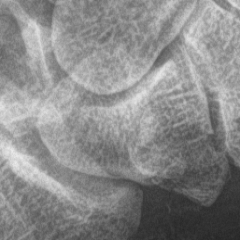

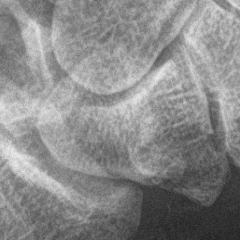

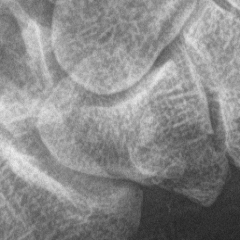

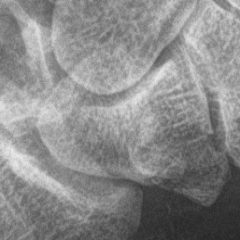


**JPEG Compression**

This is a distortion that simulates JPEG format compression, forming compression artifacts in the images.

We used *imgaug*, function ***imgaug.augmenters.arithmetic.JpegCompression*** for this process. (located at https://imgaug.readthedocs.io/en/latest/source/api_augmenters_arithmetic.html#imgaug.augmenters.arithmetic.JpegCompression) The parameter: *compression level* must be in value range [0, 100]. Here we used compression level ∈ {10, 30, 50, 70, 90}. The greater the number, the worse the image quality would be expected to be.


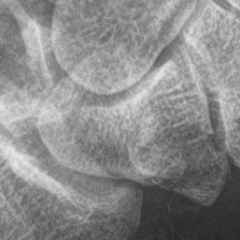


Clean dataset

JPEG Compression

compression = 10

compression = 30

compression = 50

compression = 70

compression = 90


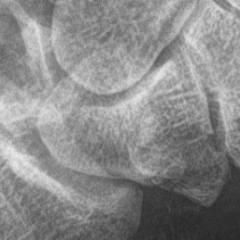

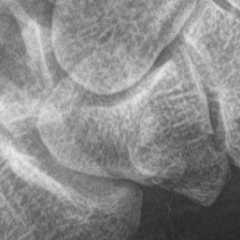

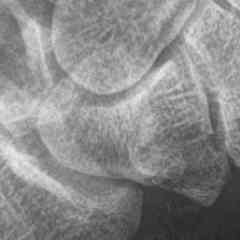

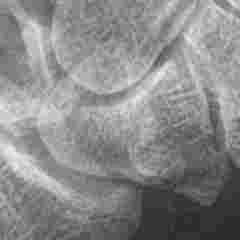

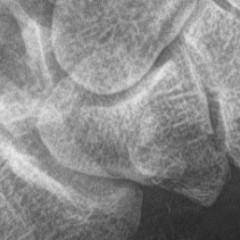


**Contrast Limited Adaptive Histogram Equalization (CLAHE)**

CLAHE [19] is a method to enhance contrast in images by histogram equalization [20]. It is a popular enhancement process applied to X-ray related tasks. Though this proposed model was not trained with CLAHE-enhanced images, its response to CLAHE was investigated because of clinical relevance.

CLAHE process was achieved with OpenCV-Python, function cv2.createCLAHE (located at https://docs.opencv.org/4.x/d6/dc7/group__imgproc__hist.html#gad689d2607b7b3889453804f414ab1018). The parameter **clipLimit**, the threshold for contrast limiting, was set to {1.0, 2.0, 3.0, 4.0, 5.0}, while **tileGridSize**, the size of grid for histogram equalization, was kept at the default (8,8)


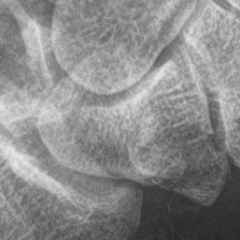


Clean dataset

CLAHE

clipLimit= 1.0

clipLimit= 2.0

clipLimit= 3.0

clipLimit= 4.0

clipLimit= 5.0


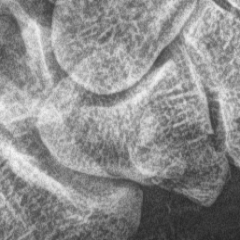

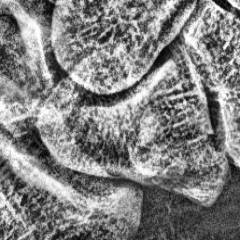

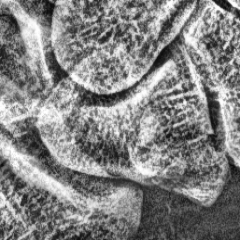

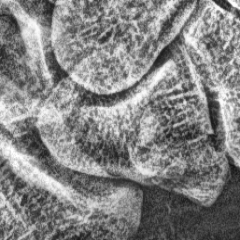

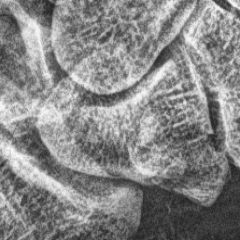


**Resizing**

Resizing an image to a smaller resolution will lead to loss of detail and blurriness.

To prepare the resized data, we resized the entire hand X-ray images to a particular resolution. The width of the image was adjusted to target width ∈ {400, 600, 800, 1000, 1200, 1400}, and the target height was calculated using: **(original height)*(target width)/(original width)** to keep the aspect ratio. Next, we mapped the bounding box label onto the new image, cropped out the scaphoid region, and resized it to 240 × 240 dimensions.

The distortion severity that results from resizing depends on the resolution of the original image. If the original resolution and target resolution are close to each other, then the effect of resizing will be small. Since the images in the dataset had various original resolutions, the extent of damage caused by resizing varied for each image.

Resizing was performed by cv2.resize (located at https://docs.opencv.org/4.x/da/d54/group__imgproc__transform.html#ga47a974309e9102f5f08231edc7e7529d), with bilinear interpolation.


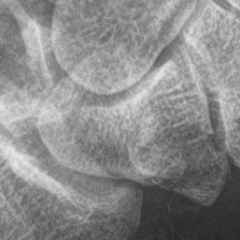


Clean dataset

Resize

width = 400

width = 600

width = 800

width = 1000

width = 1200

width = 1400


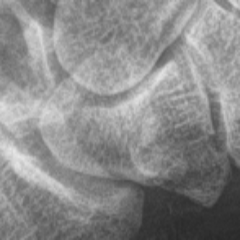

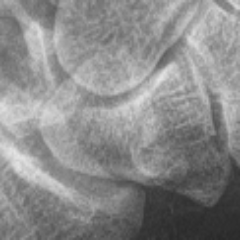

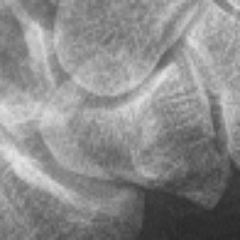

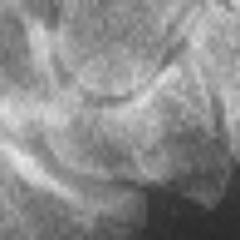

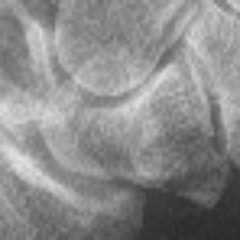

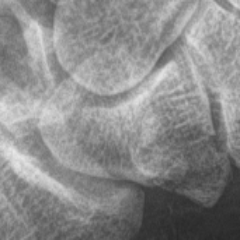


**Geometric Modification**

These 4 datasets simulated offsets in bounding boxes that could have been potentially caused by the scaphoid detector. Though the detector rarely makes mistakes, even on low-quality X-ray images, its result may still have small offsets that cause the scaphoid to appear uncentered in the bounding box. The bounding box may also have an unbalanced aspect ratio that produces distortions in scaphoid features after being cropped and resized. To simulate this situation of the detection model yielding inaccurate outputs, we modified the location of the bounding boxes. In images with the greatest modifications, some scaphoids may have been captured partially in the bounding boxes, with some portions cropped out.

**Shifting:** We shifted bounding boxes in the directions indicated by the eight points of a compass, and derived 8 scaphoid images from one X-ray image. The maximal offset was 10% or 20% of the width and height of the bounding box.

**Distortion**: Bounding boxes were composed from four values: xmin, xmax, ymin, ymax. We increased or decreased the four values by 5% or 10% of the width and height of the bounding box, forming 16 derivative scaphoid images from one X-ray image. This treatment twisted the shape of the scaphoids.


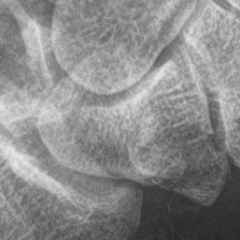


Clean dataset

Geometric Modify

Geometric_1

(moving bounding box 20%)

Geometric_2

(moving bounding box 10%)

Geometric_3

(twisting bounding box coordinates 10%)

Geometric_4

(twisting bounding box coordinates 5%)


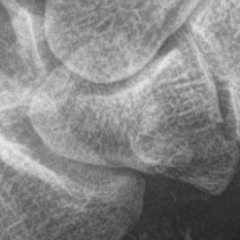

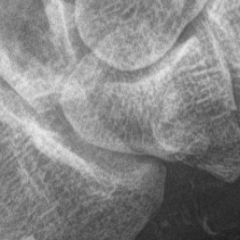

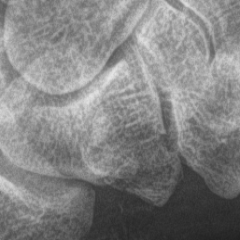

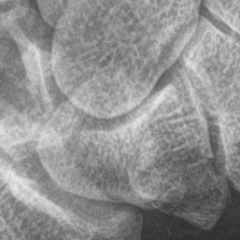


**12-bit-rescale**

We read the original DICOM files according to their bit depth (2, as 12-bit depth), and saved them into PNG formats. From these PNG images, 2 datasets were derived.

**1**. We applied the bounding box label to these images.

**2**. The images were passed to the detection model, and the marked regions were cropped out. Compared to 1, this dataset contained some interference from the detector.

Due to contrast adjustment, the images were slightly brighter or darker than the clean dataset.

These datasets did not have much noise. Despite these minor differences from the clean dataset, we sought to determine whether they could still affect model performance.


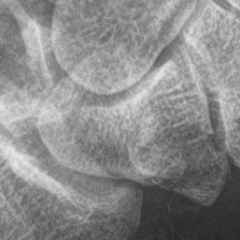


Clean dataset

12-bit Rescale

12-bit-rescale_1

12-bit-rescale_2


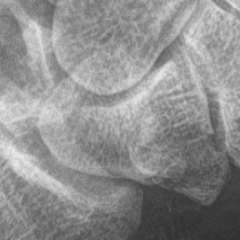

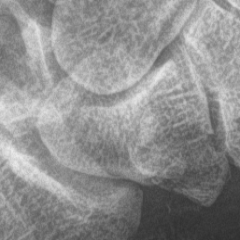


**Screenshots**

The 3 screenshot datasets were composed of screenshots collected from 2 DICOM image viewer programs. We opened the DICOM test set and took the screenshots with these programs.

Screenshot resolution depends on the display resolution. Since most X-ray images have a resolution considerably higher than normal desktop screens, image quality is heavily damaged during the screenshot process in a similar way to resizing. Moreover, there are several other factors that will influence images such as contrast, threshold, color, artifacts, and other display settings, so screenshot is the most complex of all the noise treatments. The final goal of this research was to ensure acceptable model performance under this complex condition.

Two of the datasets were both taken from MicroDicom (located at https://www.microdicom.com/), but in different scales. **Screenshot_MicroDicom_1** consisted of DICOM images viewed at default size, and the screenshot region (size: 550 × 780) included the whole hand X-ray. **Screenshot_MicroDicom_2** was similar, but in fullscreen mode and had better resolution (size: 900 × 1050).

**Screenshot_ImageJ** datasets were collected under ImageJ (located at https://imagej.net/ij/index.html). After opening the DICOM images, the view contrast display range was adjusted to 12-bit. Then, the images were resized to width = 600 pixels while keeping the aspect ratio, and screenshot. Since aspect ratio varies from image to image, the width of the images was always 600, whereas the height was not a constant. Finally, the viewing window was screenshot with resolution 585 × (height - 20). If the height exceeded 990, only the top 850 pixels were screenshot.

The screenshots contained the whole hand X-ray, but only scaphoid regions were needed. The scaphoid images were cropped and resized to 240 × 240 by the scaphoid detection model, forming the screenshot noisy datasets.

MicroDicom was running on Windows 10 with display resolution 1920 × 1080. ImageJ was running on Linux Ubuntu 18.04 with display resolution 1920 × 1080. The Python package used for screenshotting was PyAutoGUI (located at https://pyautogui.readthedocs.io/en/latest/screenshot.html#the-locate-functions).


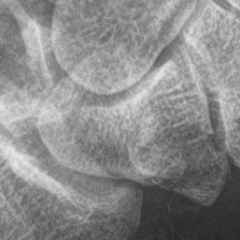


Clean dataset

Screenshots

MicroDicom

(default size)


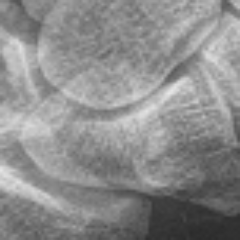

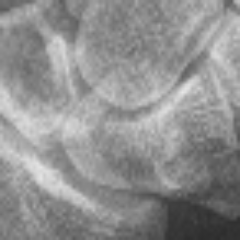

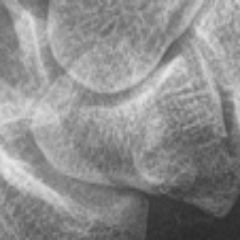


MicroDicom

(fullscreen)

ImageJ

(width = 600)

**Visual Examples and Model Confidence Across Degradation Levels**

Images of a control(non-fracture) example, with image quality metrics and model confidence. of all the distortions that PSNR and SSIM could be applied to. Though not strictly followed, while image quality drops, model confidence decreases as well. The level of decreasing, or to say the critical degradation threshold that misleads the model into a wrong decision and causes lower precision rate, varies between different kinds of treatments. The only exception from the treatments is CLAHE, as shown in Table II that CLAHE does not decrease precision rate, but increase it instead.

|  | 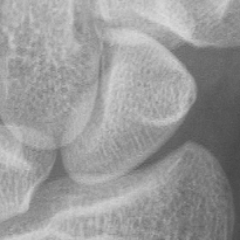 | 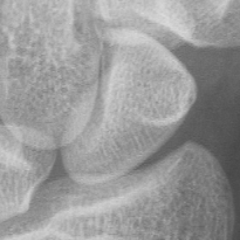 | 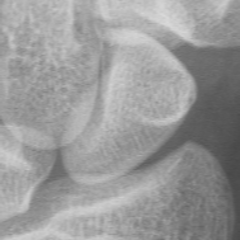 | 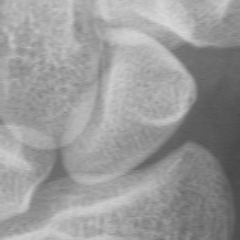 | 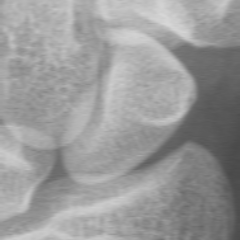 | 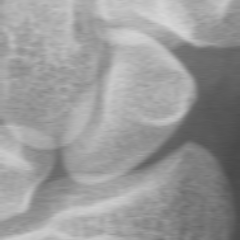 | 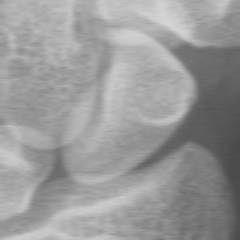 |
| --- | --- | --- | --- | --- | --- | --- | --- |
| Treatment | clean | blur_0.5 | blur_1 | blur_1.5 | blur_2 | blur_2.5 | blur_3 |
| PSNR | inf | 51.9005 | 42.4141 | 39.7376 | 37.3253 | 35.9042 | 35.3339 |
| SSIM | 100. | 0.9968 | 0.9705 | 0.9449 | 0.9062 | 0.8746 | 0.8588 |
| Confidence | 0.9987 | 0.9900 | 0.7178 | 0.9120 | 0.4730 | 0.0312 | 0.0238 |

|  | 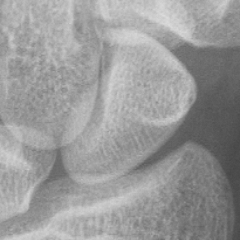 | 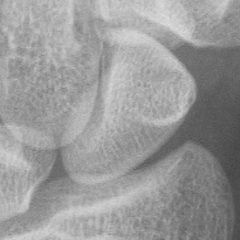 | 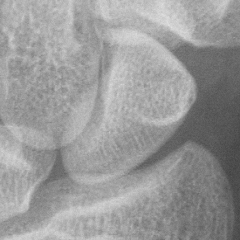 | 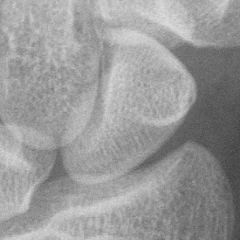 | 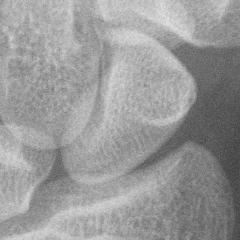 | 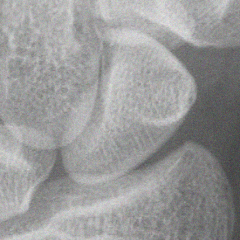 |
| --- | --- | --- | --- | --- | --- | --- |
| Treatment | noise(grayscale)_1 | noise(grayscale)_2 | noise(grayscale)_3 | noise(grayscale)_4 | noise(grayscale)_5 | noise(grayscale)_6 |
| PSNR | 47.7738 | 42.0707 | 38.5399 | 36.0101 | 34.1268 | 32.5494 |
| SSIM | 0.9912 | 0.9681 | 0.9309 | 0.8840 | 0.8331 | 0.7785 |
| Confidence | 0.9996 | 0.9993 | 0.8853 | 0.9349 | 0.7097 | 0.6173 |

|  | 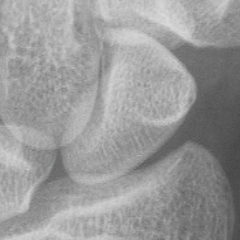 | 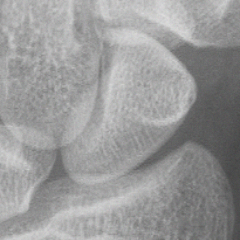 | 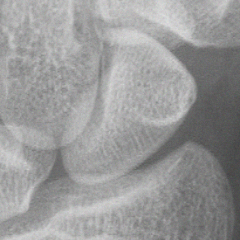 | 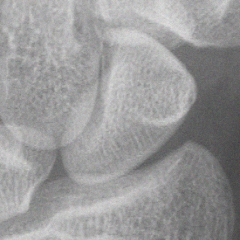 | 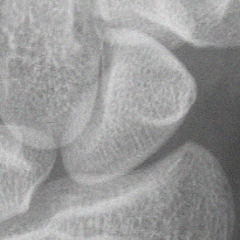 | 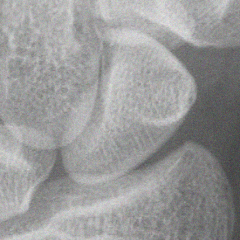 |
| --- | --- | --- | --- | --- | --- | --- |
| Treatment | noise(RGB)_1 | noise(RGB)_2 | noise(RGB)_3 | noise(RGB)_4 | noise(RGB)_5 | noise(RGB)_6 |
| PSNR | 47.7904 | 42.0381 | 38.5481 | 36.0980 | 34.1485 | 32.5494 |
| SSIM | 0.9911 | 0.9677 | 0.9312 | 0.8863 | 0.8340 | 0.7785 |
| Confidence | 0.9994 | 0.9947 | 0.9944 | 0.9659 | 0.2866 | 0.0222 |

|  | 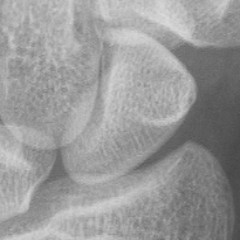 | 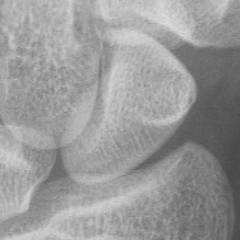 | 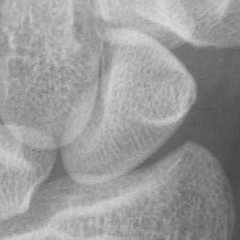 | 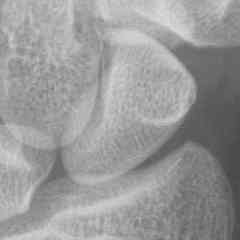 | 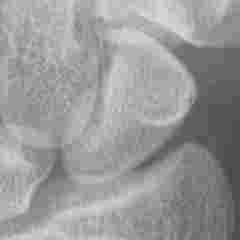 |  |
| --- | --- | --- | --- | --- | --- | --- |
| Treatment | JPEG compression_10 | JPEG compression_30 | JPEG compression_50 | JPEG compression_70 | JPEG compression_90 |  |
| PSNR | 43.4115 | 39.9295 | 38.0747 | 36.3762 | 32.8481 |  |
| SSIM | 0.9759 | 0.94761 | 0.9224 | 0.8900 | 0.7899 |  |
| Confidence | 0.9774 | 0.9814 | 0.9999 | 0.8016 | 0.0098 |  |

|  | 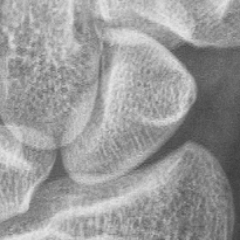 | 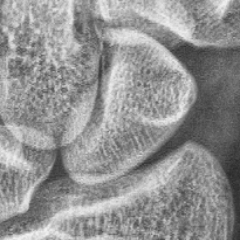 | 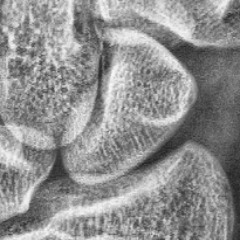 | 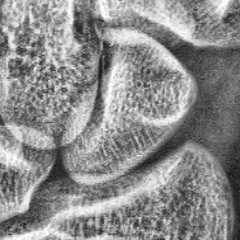 | 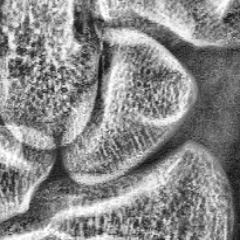 |  |
| --- | --- | --- | --- | --- | --- | --- |
| Treatment | CLAHE_1 | CLAHE_2 | CLAHE_3 | CLAHE_4 | CLAHE_5 |  |
| PSNR | 29.1177 | 21.9906 | 19.7091 | 17.3726 | 16.1140 |  |
| SSIM | 0.9276 | 0.7710 | 0.6703 | 0.5681 | 0.5116 |  |
| Confidence | 1.000 | 1.000 | 1.000 | 0.9999 | 0.9984 |  |

|  | 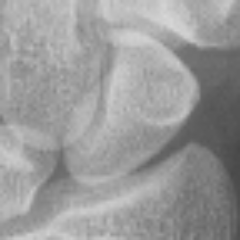 | 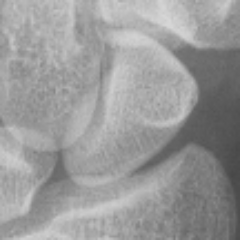 | 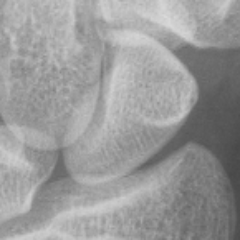 | 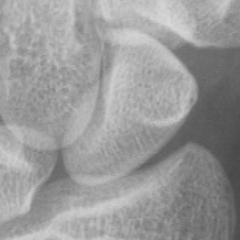 | 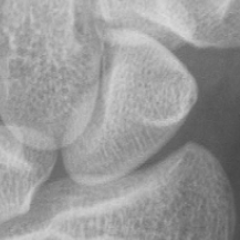 | 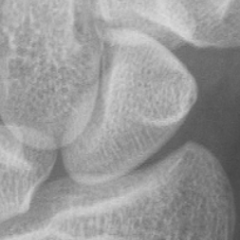 |
| --- | --- | --- | --- | --- | --- | --- |
| Treatment | Resize_400 | Resize_600 | Resize_800 | Resize_1000 | Resize_1200 | Resize_1400 |
| PSNR | 30.0205 | 30.4448 | 31.2837 | 32.6738 | 34.5699 | 38.2976 |
| SSIM | 0.7118 | 0.7264 | 0.7407 | 0.8085 | 0.8668 | 0.9389 |
| Confidence | 0.0040 | 0.9230 | 0.9975 | 0.9991 | 0.9987 | 0.9682 |
